# Cost-effectiveness of the treatment of uncomplicated severe acute malnutrition by community health workers compared to treatment provided at an outpatient facility in rural Mali

**DOI:** 10.1186/s12960-018-0273-0

**Published:** 2018-02-20

**Authors:** Eleanor Rogers, Karen Martínez, Jose Luis Alvarez Morán, Franck G. B. Alé, Pilar Charle, Saul Guerrero, Chloe Puett

**Affiliations:** 1 Action Against Hunger UK, First Floor - Rear Premises - 161-163 Greenwich High Road - London, London, SE10 8JA UK; 2 Action Against Hunger USA, One Whitehall St, New York, NY 10004 USA; 3 Area of Preventive Medicine and Public Health, Rey Juan Carlos University, Avda. Atenas s/n, 28922 Alcorcón (Madrid), Spain; 4 Fundación Acción Contra el Hambre | ACF-Spain, C/ del Duque de Sevilla, 328002 Madrid, Spain

**Keywords:** Severe acute malnutrition (SAM), Community health workers (CHWs), Community-based Management of Acute Malnutrition (CMAM), Cost-effectiveness, Mali

## Abstract

**Background:**

The Malian Nutrition Division of the Ministry of Health and Action Against Hunger tested the feasibility of integrating treatment of severe acute malnutrition (SAM) into the existing Integrated Community Case Management package delivered by community health workers (CHWs). This study assessed costs and cost-effectiveness of CHW-delivered care compared to outpatient facility-based care.

**Methods:**

Activity-based costing methods were used, and a societal perspective employed to include all relevant costs incurred by institutions, beneficiaries and communities. The intervention and control arm enrolled different numbers of children so a modelled scenario sensitivity analysis was conducted to assess the cost-effectiveness of the two arms, assuming equal numbers of children enrolled.

**Results:**

In the base case, with unequal numbers of children in each arm, for CHW-delivered care, the cost per child treated was 244 USD and cost per child recovered was 259 USD. Outpatient facility-based care was less cost-effective at 442 USD per child and 501 USD per child recovered. The conclusions of the analysis changed in the modelled scenario sensitivity analysis, with outpatient facility-based care being marginally more cost-effective (cost per child treated is 188 USD, cost per child recovered is 214 USD), compared to CHW-delivered care. This suggests that achieving good coverage is a key factor influencing cost-effectiveness of CHWs delivering treatment for SAM in this setting. Per week of treatment, households receiving CHW-delivered care spent half of the time receiving treatment and three times less money compared with those receiving treatment from the outpatient facility.

**Conclusions:**

This study supports existing evidence that the delivery of treatment by CHWs is a cost-effective intervention, provided that good coverage is achieved. A major benefit of this strategy was the lower cost incurred by the beneficiary household when treatment is available in the community. Further research is needed on the implementation costs that would be incurred by the government to increase the operability of these results.

**Electronic supplementary material:**

The online version of this article (10.1186/s12960-018-0273-0) contains supplementary material, which is available to authorized users.

## Background

Acute malnutrition accounts for over 11% of under-5 deaths globally, of which 540,000 per year are attributed to severe acute malnutrition (SAM) alone [1]. Historically, SAM was treated in inpatient facilities, an approach requiring hospital beds, highly trained medical staff and carers to accompany their child during treatment [2]. The Community-based Management of Acute Malnutrition (CMAM) model was developed to address the resource requirements of inpatient care and has been integrated into health systems of over 70 national governments [3]. The CMAM model, relying primarily on the domiciliary use of Ready to Use Therapeutic Foods (RUTF), has not only increased service coverage relative to inpatient treatment while delivering comparable clinical outcomes, but also has reduced the costs of care. In Ethiopia, the cost per child treated in inpatient care was 285 USD compared with 135 USD for community-based care [4]. Other studies found the cost-effectiveness of the approach to be comparable with other child health interventions, costing between 42 USD and 53 USD per disability-adjusted life year (DALY) averted [5, 6]. From a societal perspective, the comparative cost-effectiveness is even more apparent, with one study reporting household costs to be 6 USD (rounded) per child treated compared with 21 USD (rounded) for inpatient care [4].

Despite this economic and clinical success, coverage of services taken to scale is regularly reported to be below 50%. This is largely due to barriers including high costs for travel and opportunity costs of time required of beneficiaries [7–9].

Influenced by the success of Integrated Community Case Management (iCCM), a handful of programmes have employed community health workers (CHWs) to deliver SAM treatment, relieving beneficiaries of visits to health facilities. Trials in Bangladesh and Ethiopia found CHWs were able to provide acceptable levels of care [10, 11]. In Bangladesh, CHWs delivered high-quality care [11, 12] despite multiple barriers [13] and without sacrificing quality of other tasks [14]. A variation of these models has been used in Malawi, South Sudan and Angola [15–17], with recovery rates over 85%. Data from Bangladesh found this approach to be cost-effective, costing 26 USD per DALY averted and reducing households’ financial burden [18].

To further understand costs to both providers and beneficiaries, between 2015 and 2016, the Nutrition Division of the Ministry of Health of Mali and Action Against Hunger tested the feasibility of integrating SAM treatment into the existing iCCM package delivered by CHWs. The objectives of the present study were to estimate costs of CHW-delivered care (intervention arm) and outpatient facility-based care (control arm) from a societal perspective and to assess the cost-effectiveness of both interventions.

## Methods

### Study context

A prospective multicentre clinical cohort trial was conducted over 12 months to assess treatment of uncomplicated SAM. In the intervention arm, 18 CHWs screened for SAM, referred complicated cases, treated uncomplicated cases in communities and provided nutrition information sessions to communities. In this arm, three outpatient health facilities continued to manage cases of SAM. In the control arm, 16 CHWs screened and performed nutrition sensitisation, referring all cases to the outpatient facility for treatment or referral to inpatient care, as per the existing Malian CMAM protocol. Community health volunteers also screened and referred cases of acute malnutrition in both arms. The Ministry of Health provided treatment while Action Against Hunger supervised activities. UNICEF provided RUTF and the United States Agency for International Development and Action Against Hunger paid a portion of CHWs’ salaries, as per existing agreements. Selection criteria were as follows: child classified as SAM according to national protocol (i.e. aged 6 to 59 months; middle upper arm circumference < 115 mm; bilateral oedema or weight for height Z-score < − 3) and parental consent obtained. Trial findings have been reported elsewhere [19].

### Analytical strategy

Costs were calculated using both accounting records and information obtained via key informant interviews with staff, partners, community leaders and beneficiaries. An activity-based cost analysis was developed, and time allocation interviews were conducted, to assign costs to programme activities. A societal perspective was adopted to include all costs incurred by institutions, beneficiaries and communities. Costs were adjusted for inflation using a Consumer Price Index, converted into US dollars and presented in 2016 USD. Effectiveness data was collected as part of the trial, and cost-effectiveness ratios were calculated for cost per child recovered. Univariate and multivariate sensitivity analyses were conducted, along with a modelled scenario assuming equal numbers of enrolled children in each arm.

### Data collection

Field data was collected in January 2016. Costs were estimated via document review, interviews (*n* = 59) and focus group discussions (FGDs; *n* = 10, 5 per arm). FGDs were conducted in villages purposively selected to account for health system, demographic and geographic characteristics. Sixty-eight carers, with a child in treatment or recently exited, discussed their travel time, costs and local wage rates, to estimate forgone income.

Outcome data was obtained from the cohort trial (Table 1), with recovery rate being the primary outcome used in this study. A ‘defaulter’ is classified as a child that misses two consecutive weigh-ins, either at the outpatient therapeutic centre (OTP) for the control group or with the CHW in the intervention group. A non-responder is a child that fails to respond to treatment after 12 weeks, including referral to inpatient care and a treatable cause cannot be found.

**Table 1:** Cohort outcomes

	Intervention		Control	
Outcome	Number	Percent	Number	Percent
Recovered	581	94.17	187	88.21
Defaulted	28	4.54	23	10.85
Dead	5	0.81	2	0.94
Non-responder	3	0.49	0	0.00
Total discharged	617	100	212	100

The proportion of each cohort that died during the study was similar (0.8% exits in the intervention group compared to 0.9% in the control) and was found to have no significant difference (*p* value = 0.99). There is no reason to believe that a child’s inclusion in the control group would have increased their chance of survival.

### Data analysis

#### Activity-based cost analysis

Eight key cost centres were identified to which costs were allocated as described in Table 2.

**Table 2:** Cost and time allocation source information per cost centre

Activity	Description	Data sources
Support	Non-governmental organisation (NGO) finance, human resources and logistics services, NGO office and staff accommodation, transport (motorbikes, fuel and vehicle repairs), office materials, health centre rent and utilities, opportunity costs of community leaders time and rent for community-based health centre where CHW-delivered services in intervention arm.	Review of NGO accounting data, time allocation interviews with government and NGO field and management staff, government costs estimated through interviews with management staff. Community time and missed labour costs estimated through time allocation interviews with key informants from community and cross checked with NGO and government staff.
Supervision and monitoring	Salaries: field supervisors and monitors, NGO management staff, government supervision staff, costs associated with CHW monthly meetings.	Review of NGO accounting data, ‘off budget’ costs for government staff estimated through interviews, time allocation interviews with government and NGO field and management staff.
Training	Salaries: field staff, NGO management staff, CHWs in intervention and control arms. Direct training costs: location, trainers, transport and per diems.	Review of NGO accounting data, ‘off budget’ costs for government staff estimated through interviews, time allocation interviews with government and NGO field and management staff.
Sensitisation and mobilisation	Salaries: field supervisors, monitors, CHWs for intervention and control arms. Direct mobilisation activity costs.	Review of NGO accounting data, ‘off budget’ costs estimated through interviews with government and NGO partners, time allocation interviews with field supervisors and monitors.
Screening	Salaries: CHWs intervention and control and CHW volunteers	Review of NGO accounting data, ‘off budget’ costs estimated through interviews with government and NGO partners. Time allocation interviews with field and management staff.
Counselling	Salaries: CHWs intervention and control and CHW volunteers.	Review of NGO accounting data, ‘off budget’ costs estimated through interviews with government and partners. Time allocation interviews with field and management staff.
Treatment	RUTF (supply, transport and storage), salaries: CHWs intervention arm and health centre staff, NGO office and accommodation, implementation materials (scales, bowls and spoons, sugar for appetite test, hygiene products), storage.	Review of NGO accounting data, interviews with UNICEF, Save the Children, Action Against Hunger and government staff, time allocation interviews with Action Against Hunger and government staff.
Household costs	Opportunity costs of accessing treatment and money spent accessing services	Focus group discussions with beneficiary households on time allocated to accessing treatment, financial costs and lost income.

#### Cost-effectiveness analysis, base case

Intervention and control arms were analysed in TreeAge Pro 2016 software using separate models comparing costs and outcomes to a ‘do nothing’ alternative; this assumes zero costs and no recovery of children from SAM due to limited evidence on recovery rates in absence of treatment. These models isolate the costs and effects of both arms, to assess how they performed independently. Incremental analysis is standard when comparing cost-effectiveness of an intervention with its next-best alternative; however, the method assumes a similarity of structure in the two interventions under comparison, for example, adding an additional component to an existing intervention. Such a comparative model was not considered relevant for comparing CHWs with outpatient facility-based care; these are not incremental programs per se but require a change in service delivery infrastructure.

Several sensitivity analyses were conducted to determine whether plausible variations in costs and outcomes would have resulted in a significant change in which service was determined to be most cost-effective.

#### Sensitivity analyses

##### Modelled scenario sensitivity analysis

Multiple challenges were encountered in reaching the sample size in the control arm, including a suspected lower than anticipated prevalence of SAM, and possible researcher bias favouring enrolment in the intervention arm. The study team considered intervening to increase the number of enrolled children but decided that by doing so, the integrity of the comparison of the two service delivery models would have been compromised as access to each was a key variable under study. This posed challenges in assessing relative cost-effectiveness and was addressed by conducting a scenario sensitivity analysis, modelling equal number of children treated in each arm using the sample size in the intervention area (*n* = 617). This allowed assessment of both approaches assuming equal availability and accessibility of services.

For modelling, all costs were categorised as fixed or variable. Fixed costs in the control arm were assumed adequate to cover over 600 children as this was feasible with similar resources in the intervention arm. Variable costs per child, including costs to households, and the cost of RUTF purchase, storage, security and transport were multiplied by the total number of beneficiaries to estimate a total cost. Recovery rates in both arms are equal to the base case.

This scenario serves as an alternative base case and was subjected to all sensitivity analyses. Results are summarised in the ‘Results’ section and presented in Additional file 1.

##### Univariate sensitivity analysis

Univariate sensitivity analyses were conducted, varying each parameter individually across a range of plausible values. Plausible variation for costs was based on the study team’s estimates of potential variation, and calculated as a maximum and minimum cost per child per study arm. The base case recovery rate in the intervention arm of 94.17% was considered as a reasonable maximum possible rate. A plausible minimum rate was calculated by assuming as non-recovered all children transferred to other facilities and lost to follow-up, the outcomes for which were not tracked in the cohort study, as shown in Table 3.

**Table 3:** Recovery rate, worst-case scenario

Outcome	Intervention		Control	
	Number	Percent	Number	Percent
Recovered	581	83.12	187	79.57
Defaulted	28	4.01	23	9.79
Dead	5	0.72	2	0.85
Non-responder	3	0.43	0	0.00
Transferred to inpatient/other outpatient facility	81	11.59	22	9.36
Lost to follow-up	1	0.14	1	0.43
Total	699	100	235	100

##### Multivariate sensitivity analysis

Multivariate probabilistic sensitivity analyses were conducted to assess joint variation in all parameters, using 100 000 iterations per model. Gamma distributions were used to characterise cost parameters and beta distributions for recovery rates.

## Results

### Costs

In the base case, the total cost was 150 523 USD for the intervention arm and 93 641 USD for the control, as presented in Table 4.

**Table 4:** Project input costs by arm, base case

	Intervention		Control	
All costs	USD	% total costs	USD	% total costs
*Personnel*	*87 926*	*58.4*	*56 632*	*60.5*
CHWs (salaries and incentives)	10 332	6.9	3 982	4.3
Field supervision and medical staff	28 193	18.7	17 194	18.4
Management and technical staff	45 656	30.3	32 536	34.7
Support staff (logistics, finance, administrative)	3 746	2.5	2 921	3.1
*Programme costs*	*35 648*	*23.7*	*20 848*	*22.3*
Office and programme materials	1 756	1.2	1 324	1.4
RUTF (supply)	17 795	11.8	5 648	6.0
Training costs (trainer, location, supplies)	9 248	6.1	7 407	7.9
Supervision and monitoring	6 849	4.6	6 469	6.9
*Logistics*	*20 732*	*13.8*	*12 953*	*13.8*
Rent and utilities	8 927	5.9	2 767	3.0
Transport (car rental, maintenance and fuel)	10 025	6.7	9 621	10.3
RUTF transport and storage	1 780	1.2	565	0.6
*Community contributions*	*6 218*	*4.1*	*3 208*	*3.4*
Costs to households	4 807	3.2	3 191	3.4
Opportunity costs for community leaders	35	0.0	17	0.0
Community-level rent	1 376	0.9	0	0.0
*Total*	*150 523*	*100.0*	*93 641*	*100.0*
Cost to government	11 881	7.9	5 341	5.7
Cost to partners	132 425	88.0	85 093	90.9
Cost to community	6 218	4.1	3 208	3.4

Italics indicate input cost categories, with related figures summarising the cost and the proportion of the total cost of each category

Costs in the intervention arm were higher than those in the control, although the proportion of total costs in each input category was similar. CHW costs were nearly three times higher in the intervention arm compared to the control, reflecting their increased role in service delivery. Costs to beneficiaries were higher in the intervention arm due to higher enrolment, but individual households spent less time and money receiving treatment. On average per week of treatment, households receiving CHW-delivered care spent nearly half the amount of time receiving treatment and three times less money compared with the outpatient facility-based arm (2.15 h versus 3.92 h; 0.60 USD versus 1.70 USD). These higher costs were due to transportation to the facility, a cost not incurred by those who received CHW-delivered care within a 10-min walk from home.

**Fig. 1:**
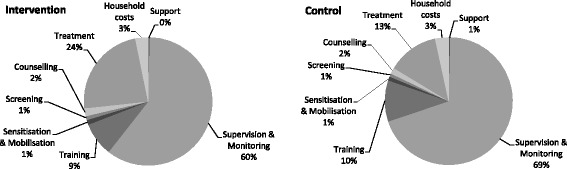
Activity-based costs for the intervention and control arms, base case

Cost proportions, presented in Fig. 1, were similar across both arms, excepting treatment costs. Supervision and monitoring was the most costly activity with the majority of these costs in each arm attributed to the salary of an expatriate research coordinator (40%) and five CHW supervisors (33%) and associated vehicle rental; staff salaries were adjusted to exclude time spent on research activities. The remaining costs of this activity were a proportion of government and non-governmental organisation (NGO) staff salaries and meeting costs from the local to the national levels. Treatment costs were higher for the intervention arm primarily because of the increased cost of RUTF associated with treating more children.

### Cost-effectiveness

In the base case with the observed number of children treated, the average cost per child treated by CHWs was 244 USD compared to 442 USD in the outpatient facility as shown in Table 5. The cost per child recovered was 259 USD by CHWs and 501 USD in the outpatient facility. The base case analysis shows outpatient facility-based care to be considerably more expensive than CHW-delivered care.

**Table 5:** Base case and modelled scenario cost-effectiveness results

Outcome	Intervention (observed, base case)	Control (observed, base case)	Intervention (modelled scenario)	Control (modelled scenario*)
Total cost (USD)	150 523	93 641	146 744	116 251
# children in program	617	212	617	617
Recovery rate	94.17%	88.21%	94.17%	88.21%
Number of children recovered	581	187	581	544^a^
Cost per child treated	244	442	238	188
Cost per child recovered	259	501	253	214

^a^Modelled number based on sample size of 617

*Number of children admitted is modelled to be the same despite initial unequal arms

### Sensitivity analysis

Parameter values and ranges are presented in Table 6.

**Table 6:** Model parameter values and ranges

Parameter	Base case	Worst case	Best case	Source
Recovery rate, intervention	94.17%	83.12%	94.17%	Base—cohort study Worst—percentage including transfers and loss to follow-up Best—base case for intervention arm
Recovery rate, control	88.21%	79.57%	94.17%	
Cost per child, intervention, base case	244	260	228	Base—average cost per child Worst/Best—calculated based on percentage range for each cost sub-category
Cost per child, control, base case	442	460	424	
Cost per child, intervention, modelled scenario	238	269	166	
Cost per child, control, modelled scenario	188	216	133	

#### Univariate sensitivity analysis

The univariate sensitivity analysis found that, in the base case in the intervention arm, both cost and recovery variables had similar levels of uncertainty (analysis not shown). Variation in recovery rate changed the cost per case recovered from 259 to 293 USD. In the control arm, the recovery rate variable showed higher sensitivity, with results ranging from 469 to 555 USD. The cost variable had a narrower uncertainty range, from 480 to 521 USD. These results indicate that without adjusting the number of children treated (as was done in the modelled scenario), outpatient facility-based care is more expensive than CHW-delivered care, even when accounting for plausible variation in model inputs.

#### Multivariate probabilistic sensitivity analysis

**Fig. 2:**
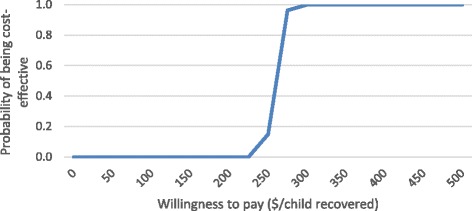
Acceptability curve—CHW-delivered care, base case

Acceptability curves indicate the probability that the program would achieve various costs per child recovered. Figure 2 shows that in the base case for CHW-delivered care, the probability that the intervention would be cost-effective is 25, 50 and 75% at a willingness to pay of 253, 261 and 268 USD per child recovered respectively. The mean cost per child recovered of 259 USD had a 95% confidence interval (CI) of 242 to 277 USD.

**Fig. 3:**
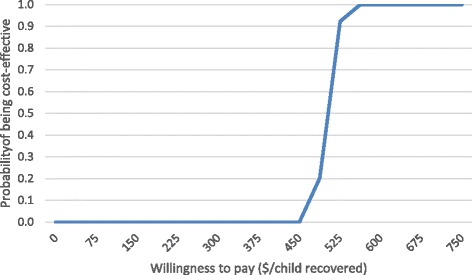
Acceptability curve—outpatient facility-based care, base case

Figure 3 shows that in the base case, the probability that outpatient facility-based care would be cost-effective is 25, 50 and 75% at a willingness to pay of 490, 503 and 516 USD per child recovered, respectively. The mean cost per child recovered in the intervention arm of 501 USD had a 95% CI of 471 to 535 USD.

#### Modelled scenario sensitivity analysis

Results for the modelled scenario sensitivity analysis are summarised in Table 5. Total program costs were 146 744 USD for the intervention and 116 251 USD for the control arm. The average cost per child treated was 238 USD by CHWs and 188 USD in the outpatient facility. The cost per child recovered was 253 USD and 214 USD in each arm respectively. Under this scenario, where each arm treated the same number of children, the cost of care is lower for outpatient facility-based care than CHW-delivered care.

Univariate sensitivity analyses show a difference in range of costs per child in the control arm between the modelled scenario (range 151 to 245) and the base case (range 469 to 555). Results for probabilistic sensitivity analyses in the control arm also differed strongly between the base case and modelled scenario, with a confidence interval of 170 to 263 USD in the scenario versus 471 to 535 USD in the base case. Taken together, these findings indicate that the level of coverage achieved by these programs, which the modelled scenario was conducted to assess, is a fundamental determinant of their cost-effectiveness. This is due in part to fixed costs being spread out over a higher number of beneficiaries when coverage is increased.

Full results from the scenario sensitivity analysis are included in Additional file 1.

## Discussion

### Cost-effectiveness

This analysis shows that community-based treatment of SAM by CHWs in Mali bears a cost per child treated and recovered consistent with results from similar analyses. A program in Bangladesh where CHWs treated SAM in communities bore a cost per child treated of 165 USD and per child recovered of 180 USD [18]. Treatment in Mali was around 50% higher, due in part to inclusion of costs of three outpatient facilities remaining open in the intervention arm. Further direct comparison is complicated by different country-level cost structures [20].

Treatment in outpatient facility-based care in the base case was more costly than any other CMAM program assessed in published literature. Results from the modelled scenario, conducted to correct for low enrolment and overcome potential bias in the inclusion of children, are in line with other CMAM programs (see Table 7 for comparisons).

**Table 7:** Available evidence of cost-effectiveness of CHW-delivered treatment and CMAM

Cost outcome	Mali				Bangladesh [18]	Ethiopia [4]	Malawi [5]	Zambia [6]
CHW/OTP	CHW	OTP	CHW	OTP	CHW	OTP	OTP	OTP
Scenario/base case	Base case	Base case	Scenario	Scenario				
Per recovered case (USD)	259	501	253	214	180	145	185	–
Per treated case (USD)	244	442	238	188	165	135	169	203

This analysis found the cost-effectiveness of SAM treatment in Mali to depend on the capacity of services to achieve high levels of coverage. While the modelled scenario illustrates the potential cost-effectiveness of facility-based services, the low enrolment in the facility-based control arm reflects, in part, the challenges and barriers associated with facility-based CMAM in Mali and other countries [7, 8, 21]. The higher admissions for CHW-delivered care suggest that a well-functioning, decentralised treatment model can overcome some of these barriers, increase levels of coverage and offer a more cost-effective alternative.

### Costs

Supervision and monitoring was the highest cost category at over half of the total budget. A slightly lower proportion of total costs was spent on monitoring and supervision in Bangladesh, although the costs within the activity were similar, including staff salaries and coordination meetings. This reflects the need for investment in strong supervision and support both in start-up and throughout service delivery for CHW-delivered care [22] as well as relatively higher costs for implementation in Mali [18]. Similarly, a high proportion of costs attributed to supervision have been reported in the start-up of iCCM programmes [23]. Efficiencies could be gained over time via employment of national staff, optimisation of service delivery, integrating supervision of iCCM components and using an existing trained cadre of CHWs, which would eliminate initial training costs. However, the scale-up of these services would carry additional costs associated with supervision in a sparsely populated setting.

RUTF-related costs were 13% of the total in the intervention arm, a low proportion compared with findings from other CMAM costing exercises (range 24–43%) [4–6, 18]. As in the Bangladesh study, this can be explained by higher costs in other categories [18]. Given that UNICEF continues to lead RUTF procurement in Mali, the costs incurred by the national government if they adopted this approach could be lower than those estimated here.

This study found that weekly costs to beneficiary households for CHW-delivered care were three times lower than facility-delivered services (0.60 USD and US 1.70 USD respectively). Moreover, each visit to the CHW took half the time required for a facility visit. The relatively lower time and money costs associated with CHW-delivered care were due predominantly to the cost of transport and time required to visit the health facility. These findings suggest that reducing beneficiary costs with CHW-delivered care is likely to increase access [24].

### Limitations

This analysis has four key limitations. First, the intervention arm assessed treatment by CHWs in addition to three outpatient health facilities; it was not possible to separate outcomes and costs between the two delivery methods. However, as only 21% of admissions went to the outpatient facility, this indicates that the majority of cases were managed by CHWs in the community. Second, although it is likely that multiple inefficiencies were encountered in service delivery in this pilot project, assessing these was not a focus of this analysis. Third, no official cost data was shared by the implementing partners, a common challenge in cost assessments, so these were estimated through interviews which may have introduced imprecision into estimates to some extent. Further research on costs to the government would provide valuable guidance for integration into health systems. Fourth, due to the impossibility of blinding the study, it is possible that researcher bias may have resulted in a greater focus on the intervention arm, providing one possible explanation for the higher numbers of enrolled children in that arm. The modelled scenario sensitivity analysis was conducted to address this risk. An additional limitation relating to the overarching study is that information on other illnesses the children incurred aside from SAM was not recorded. We have no reason to believe this was an issue due to a baseline socio-economic survey demonstrating explainable differences between the two groups and the fact that a child suffering from an unrelated, significant illness would not have been included in the study. However, it is not possible to know with certainty if both groups were similar in incidence of other illnesses.

## Conclusions

This study supports existing evidence that the delivery of treatment for SAM by CHWs is cost-effective. A major benefit of this strategy was the lower cost incurred by beneficiary households with treatment in the community, removing multiple barriers to access. Although the outpatient facility-based approach had the potential to be more cost-effective by improving coverage, programmatic evidence suggests that systematically achieving this is unlikely in operational contexts like Mali. Further research is needed on the costs to the government of implementing this strategy, to increase the operability of these results by Ministries of Health both in Mali and other contexts.

## Additional file

Additional file 1: Modelled scenario—cost data and sensitivity analyses. (DOCX 114 kb)

## Abbreviations

CHWs: Community health workers
CI: Confidence interval
CMAM: Community-based Management of Acute Malnutrition
DALY: Disability-adjusted life years
iCCM: Integrated Community Case Management
NGO: Non-governmental organisation
RUTF: Ready to Use Therapeutic Food
SAM: Severe acute malnutrition
