# Supplementary material for: Cost-effectiveness of the treatment of uncomplicated severe acute malnutrition by community health workers compared to treatment provided at an outpatient facility in rural Mali
Source: Hum Resour Health. 2018 Feb 20;16:12. doi: 10.1186/s12960-018-0273-0 (PMC5819265; doi:10.1186/s12960-018-0273-0)
Supplement: Supplementary file 1 — Modelled scenario—cost data and sensitivity analyses. (DOCX 114 kb) [file 12960_2018_273_MOESM1_ESM.docx]

# Additional file 1

## Cost table, modelled scenario

The costs for the intervention and control arm were modelled for the scenario in which each arm treated an equal number of children (n=617). Fixed costs in the control arm were assumed adequate to cover over 600 children as this was feasible with similar resources in the intervention arm. Variable costs per child, including costs to households, and the cost of RUTF purchase, storage, security and transport were multiplied by 617 to estimate a total cost.

In this scenario most of the costs of CHW delivered care remain higher than outpatient facility care. However the costs to the beneficiary household are a third higher in the control arm reflecting the associated costs of travelling to the facility.

Table 5: Costs per inputs for each arm in the modelled scenario, with an equal number of children treated in each arm

|  | **Intervention** | | **Control** | |
| --- | --- | --- | --- | --- |
|  | **USD** | **% total costs** | **USD** | **% total costs** |
| **Personnel** | **86,143** | **58.7%** | **58,415** | **50.2%** |
| CHWs *(salaried and volunteer)* | 10,332 | 7.0% | 3,982 | 3.4% |
| Local technical staff | 29,030 | 19.8% | 22,085 | 19.0% |
| NGO management and technical staff | 43,036 | 29.3% | 29,427 | 25.3% |
| Support staff *(logistics, finance, administrative)* | 3,746 | 2.6% | 2,921 | 2.5% |
|  |  |  |  |  |
| **Programme costs** | **35,300** | **24.1%** | **32,648** | **28.1%** |
| Office and programme materials | 1,756 | 1.2% | 1,324 | 1.1% |
| RUTF *(supply)* | 17,448 | 11.9% | 17,448 | 15.0% |
| Training costs *(trainer, location, supplies)* | 9,248 | 6.3% | 7,407 | 6.4% |
| Supervision and monitoring | 6,849 | 4.7% | 6,469 | 5.6% |
|  |  |  |  |  |
| **Logistics** | **19,082** | **13.0%** | **15,886** | **13.7%** |
| Rent and utilities | 7,223 | 4.9% | 4,471 | 3.8% |
| Transport *(car rental, maintenance and fuel)* | 10,114 | 6.9% | 9,669 | 8.3% |
| RUTF transport and storage | 1,745 | 1.2% | 1,745 | 1.5% |
|  |  |  |  |  |
| **Community contributions:** | **6,218** | **4.2%** | **9,303** | **8.0%** |
| Costs to households | 4,807 | 3.3% | 9,286 | 8.0% |
| Opportunity costs for community leaders | 35 | 0.0% | 17 | 0.0% |
| Community-level rent | 1,376 | 0.9% | 0 | 0.0% |
| **Total** | **146,744** | **100.0%** | **116,251** | **100.0%** |
| Cost to Government | 8,394 | 5.7% | 8,827 | 7.6% |
| Cost to Partners | 132,131 | 90.0% | 98,121 | 84.4% |
| Cost to Community | 6,218 | 4.2% | 9,303 | 8.0% |

## Activity based costs, modelled scenario

The allocation of costs per activity for the modelled scenario is similar across the two arms. However, variation appears in the proportion of costs incurred by beneficiaries (opportunity and actual), which were nearly twice as high for outpatient facility based care (8%) compared with CHW delivered care (4%).

Figure D: Activity-based costs for the control arm, modelled scenario


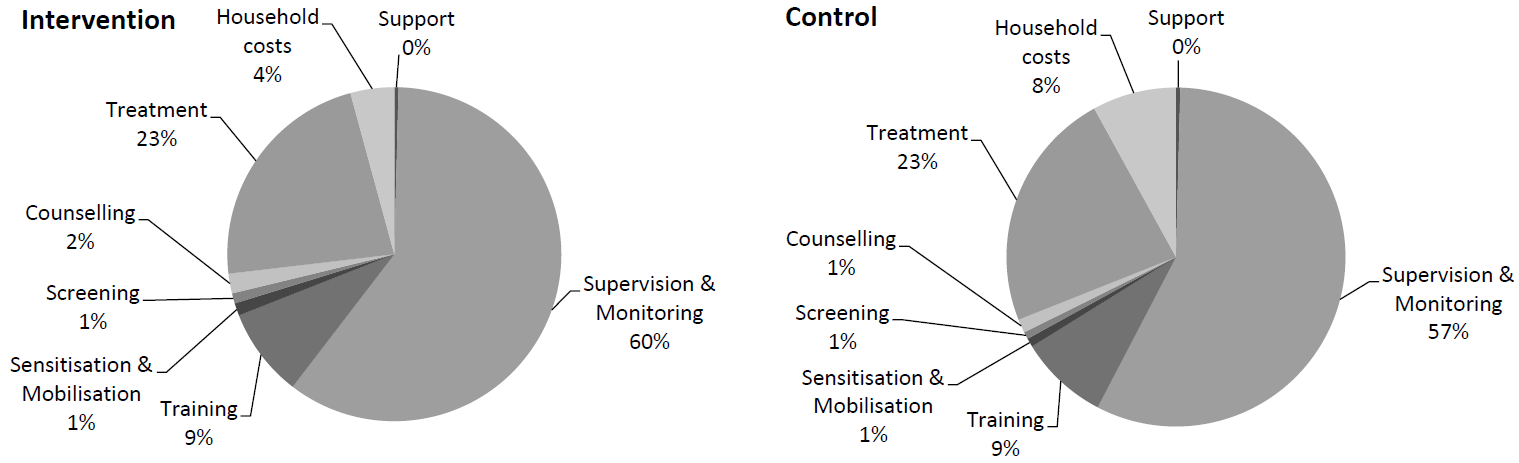


## Univariate sensitivity analysis

In univariate sensitivity analyses, the cost per child showed the highest level of uncertainty, resulting in a range in cost per case recovered of 176 to 286 USD for CHW delivered care and 151 to 245 USD for outpatient facility care. These results suggest that even taking into account plausible variation, assuming equal coverage, the cost to recover a child would not exceed 300 USD when treated by CHWs and 250 USD for outpatient facility-based care.

## Probabilistic sensitivity analysis

Figure E shows the acceptability curve from the CHW delivered care in the modelled scenario, demonstrating the probability that the intervention would be cost effective is 25%, 50% and 75% at a willingness to pay of 232, 252 and 272 USD per child recovered, respectively. The 95% CI around the mean cost per child recovered of 253 USD in this modelled scenario is 201 to 310 USD, a much larger margin than the base case.

Figure E: Acceptability curve – CHW delivered care, modelled scenario

The acceptability curve in figure F indicates that in the modelled scenario, the probability that outpatient facility-based services would be cost-effective is 25%, 50% and 75% at a willingness to pay of 196, 213 and 231 USD respectively per child recovered. The 95% CI around the mean cost per child recovered of 214 USD is 170 to 263 USD, a larger margin than the base case.

Figure F: Acceptability curve – outpatient facility-based care, modelled scenario
